# COVID-19 Vaccines: Tolerance of Vaccination in Patients with Allergies

**DOI:** 10.3390/vaccines13090904

**Published:** 2025-08-26

**Authors:** Natalie Kogseder, Viktoria Puxkandl, Wolfram Hötzenecker, Sabine Altrichter

**Affiliations:** 1 Department for Dermatology and Venerology, Kepler University Hospital, 4020 Linz, Austria; natalie.kogseder@kepleruniklinikum.at (N.K.); viktoria.puxkandl@kepleruniklinikum.at (V.P.); sabine.altrichter@kepleruniklinikum.at (S.A.); 2 Medical Faculty, Johannes Kepler University, 4020 Linz, Austria; 3 Clinical Research Institute for Inflammation Medicine, Medical Faculty, Johannes Kepler University, 4020 Linz, Austria; 4 Institute of Allergology, Charité–Universitätsmedizin Berlin, Corporate Member of Freie Universität Berlin, Humboldt-Universität zu Berlin, and Berlin Institute of Health, 12203 Berlin, Germany; 5 Fraunhofer Institute for Translational Medicine and Pharmacology ITMP, Allergology and Immunology, 12203 Berlin, Germany

**Keywords:** allergy, COVID-19, vaccine, HADS, antihistamine

## Abstract

**Background**: Due to the new COVID-19 vaccine types used in the COVID-19 vaccination program, significant levels of uncertainty and vaccine hesitancy arose due to fears about anaphylactic reactions, especially in patients with allergies. This study aimed to analyze how patients with significant allergies receiving specific immunotherapy (SIT) reacted to COVID-19 vaccines in real life. **Methods**: We assessed 57 patient charts of individuals with allergies receiving SIT for documented allergies, for their comorbidities, total IgE and tryptase levels, and Hospital Anxiety and Depression Scale (HADS) scores. Questionnaires regarding COVID-19 vaccination status and reported adverse reactions were analyzed. **Results**: Patients were more frequently male (58%) and had a mean age of 43 years, and the majority (60%) had already experienced an anaphylactic reaction in the past, most commonly to the allergen of the current SIT. In total, 93% received COVID-19 vaccinations. More than half of the patients (57%) reported at least one adverse reaction after COVID-19 vaccination. Out of these patients, 97% reacted in an unspecific way, with symptoms of general illness. One potential allergic reaction, classified as a grade 1 anaphylactic reaction, was documented. The patient with the potential allergic reaction was significantly more concerned before receiving the vaccination and had experienced possible allergic reactions to other vaccinations in the past. The remaining patients with unspecific reactions after vaccination had also experienced such adverse reactions in the past to other vaccinations. Antihistamine premedication was associated with fewer unspecific reactions after COVID-19 vaccination. **Conclusions**: Vaccinations in patients with significant allergies and prior anaphylactic reactions are, overall, safe. Prior allergic reactions after other vaccinations could be a potential risk for reaction after COVID-19 vaccinations, whereas antihistamine intake could be beneficial in reducing side effects after COVID-19 vaccinations in patients with allergies. Prospective studies on this topic are needed.

## 1. Introduction

In general, severe anaphylaxis in response to vaccinations is rare—about 1.3 people in 1 million appear to be affected [1]. Due to initial reports about anaphylactic reactions to COVID-19 vaccines shortly after the start of the initial vaccine administration, significant levels of uncertainty became apparent in the population—especially in patients with allergies [2]. Reports of people who had experienced potentially severe allergic reactions were collected in the Vaccine Adverse Event Reporting System (VAERS). They were further categorized as anaphylactic and non-anaphylactic reactions after the BioNTech/Pfizer COVID-19 vaccine, which identified 21 cases of definitive anaphylaxis after COVID-19 vaccination in December 2020 [3]. Following the VAERS, a rate of about 11.1 cases of anaphylaxis per million vaccine doses was estimated, with about 19% of patients being hospitalized and 18% receiving treatment at an emergency department [4]. The most frequently reported symptoms were any kind of rash, urticaria, angioedema, and a sense of throat swelling. The vast majority (81%) of patients with anaphylaxis had a documented history of allergies or allergic reactions, and one third had already experienced anaphylaxis in the past [3]. Therefore, at this point, the Medicines and Healthcare Products Regulatory Agency in the UK recommended excluding patients with a history of anaphylactic reactions triggered by any kind of allergens from vaccination—resulting in hesitancy in patients with allergies [2]. After reevaluation, it was announced by the European Academy of Allergy and Clinical Immunology (EAACI) that only patients with proven allergies to vaccine components meet the absolute contraindications [5]. More recent data, in fact, show a comparable rate of anaphylaxis after COVID-19 vaccine and other vaccines [6].

The suspected trigger for anaphylaxis to mRNA vaccines, such as BioNTech/Pfizer and Moderna, is polyethylene glycol (PEG)-2000 [7]. PEG is a polymer varying in size, up to 5000 g/mol. Generally, PEGs are often included in several drugs, e.g., penicillin or several laxatives—potentially stabilizing lipid nanoparticles. There have been reports of rare cases of type-I IgE-mediated allergies to PEG, especially after the administration of greater amounts of this substance [8]. Vector-based vaccines (AstraZeneca, Janssen), however, do not contain PEG2000 but instead contain polysorbate 80—equally being suspected of triggering anaphylactic reactions [1].

Several risk factors for anaphylactic reaction after COVID-19 vaccination have been reported. Macy et al. [9], for example, reported younger age, female sex, and a history of drug intolerances and prior anaphylaxis to vaccination as potential risk factors. Comparably, Shavit et al. [10] observed a higher prevalence of anaphylactic reactions in female patients with drug allergies and anaphylaxis in the past. A possible reason for this higher prevalence could be the so-called nocebo effect, which describes a reaction after the administration of an indifferent substance, occurring especially after the first dose [6].

To our knowledge, there have been no evaluations of whether patients with severe allergies receiving SIT tolerate COVID-19 vaccinations or show high rates of anaphylactic or other adverse reactions after application. To address this gap in knowledge, we aimed to analyze the following questions: (i) How did our patients with significant allergies undergoing SIT react to COVID-19 vaccinations? (ii) Can risk factors among the patients with reactions to COVID-19 vaccination be identified? (iii) Can we identify factors that differed between patients with and without adverse reactions to COVID-19 vaccinations?

## 2. Materials and Methods

### 2.1. Patients

Between September 2022 and April 2023, 57 patients presenting at the Allergy Outpatient Clinic of the Department of Dermatology and Venerology at Kepler University Hospital for routine subcutaneous or sublingual specific immunotherapy (SIT) were randomly selected for this study.

After providing informed consent, the patients completed a questionnaire.

Ethical approval was obtained from the local ethics committee (ECS No. 1152/2022). All patient records were pseudonymized in accordance with data protection and local ethics regulations.

### 2.2. Clinical Assessments

Patient data, including sex, age, prevalence of comorbidities, different types of allergies, prior anaphylaxis, laboratory parameters, and anxiety and depression scores, was obtained from patient charts and questionnaires.

The identified allergies were categorized into one of six groups: aeroallergies, contact allergies, food allergies, venom allergies, allergies to oral medication, and allergies to injectable drugs. A category was considered ‘positive’ if one or more allergies fitting into one category were documented.

Anaphylaxis was considered prevalent if the patient had previously experienced an immediate-type reaction involving dyspnea or other extracutaneous symptoms that could be categorized as ≥grade 2 anaphylactic reactions according to Ring and Messmer [11].

Concomitant allergic diseases such as asthma, atopic dermatitis, allergic rhinitis, and urticaria were identified from the patient charts. Mastocytosis was defined as prevalent if tryptase levels were >11.4 μg/L and confirmed by other diagnostic measures [12].

Patient characteristics are shown in Table 1.

### 2.3. Laboratory Assessments

The ImmunoCAP System^®^ (Phadia Laboratory Systems, Thermo Fisher Scientific Inc, Uppsala, Sweden) was used to assess total IgE and tryptase serum levels in the nuclear laboratory of the clinic as a baseline measurement in patients with no acute symptoms. A total IgE level of >112 IU/mL and a tryptase level of >11.4 μg/L were considered elevated.

### 2.4. Questionnaire

After providing their informed consent, patients completed a questionnaire about their COVID-19 vaccination status. The number of vaccinations received and the type of vaccination were evaluated. Vaccines were categorized into BioNTech/Pfizer, Moderna, Astra Zeneca, and Janssen product vaccines. If patients had received more than one type of vaccine, each type of vaccine was considered individually.

Vaccine reactions were classified into three categories: no reaction; an unspecific reaction, including general symptoms of illness such as fever, shivering, fatigue, muscle pain, limb pain, headache, or local reactions around the injection site; and a possible allergic reaction, including systemic reactions such as pruritus, urticaria, flushing, angioedema, vomiting, dyspnea, and shock of all magnitudes. All documented reactions appeared within 48 hours after vaccination. Reactions to other vaccines (e.g., influenza) were classified in the same way.

### 2.5. Psychological Assessment

The level of concern reported before receiving each vaccination dose was averaged and then divided into five categories (0–4): no concerns, mild concerns, mild/moderate concerns, moderate concerns, and severe concerns.

The Hospital Anxiety and Depression Scale (HADS) was used to screen for anxiety and depression, comprising two subscales: HADS—Anxiety (HADS-A) and HADS—Depression (HADS-D). Each subscale includes seven questions about the patient’s mental state. Up to 3 points can be awarded per question, resulting in a maximum of 21 points per subscale. A score of 0–7 is not considered clinically significant, 8–10 is considered doubtful, and 11 or more is defined as a definitely clinically significant case [13].

### 2.6. Statistical Analyses

Statistical analyses were performed using SPSS (Version 29.0.0.0 and Version 30.0.0.0). The normality test (Kolmogorov–Smirnov and Shapiro–Wilk Test) could only confirm a normal distribution for anxiety in both groups, anxiety in group hymenoptera allergies and group aeroallergies, age in group hymenoptera allergies, concerns in group aeroallergie, and depression in group aeroallergies. Statistical analysis was performed using the Mann–Whitney U test and t-test for group comparison. Spearman rank correlation tests were used to calculate correlations, and the correlation coefficients are displayed as “r”. A Chi-square test or Fisher Exact test was used for binominal variables with small categorical numbers (<5). A *p*-value of less than 0.05 was considered to indicate statistical significance, while a *p*-value of less than 0.1 was considered to show a trend.

## 3. Results

### 3.1. Most Patients Who Underwent SIT (93%) Received COVID-19 Vaccination; Among Those, 87% Received at Least One Dose of BioNTech/Pfizer Vaccine

Patients with allergies who presented for SIT were slightly more likely to be male (58%), with a mean age of 43 years. The vast majority had symptomatic allergies from only one allergen category (median 1.0, mean 1.4; see Table 1), with the most common being hymenoptera venom allergies (54%) and aeroallergies (46%). Almost 60% had already experienced anaphylaxis in the past (see Table 1). Three patients (5.3%) had a comorbid mastocytosis.

The majority of our patients (93%) was vaccinated against COVID-19. Patients who did not receive the vaccination were all allergic to hymenoptera venom but were not otherwise clinically different (see Table A1). There was also no indication of higher levels of anxiety or depression in patients who did not receive a vaccine (see Table A1). Whether and why the others were not vaccinated were not investigated further.

**Figure 1:**
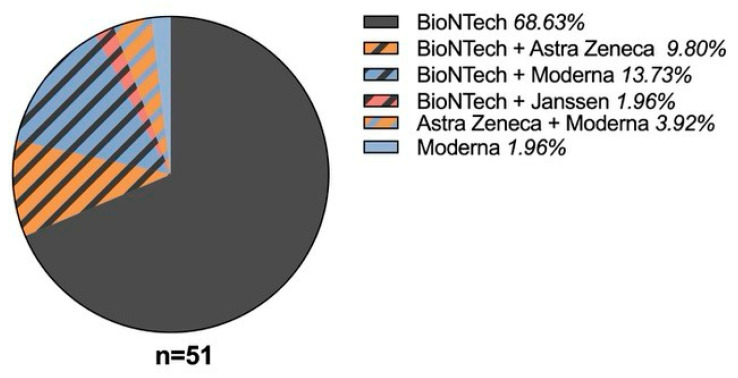
Received types of vaccination combinations in vaccinated patients with SIT.

**Figure A1:**
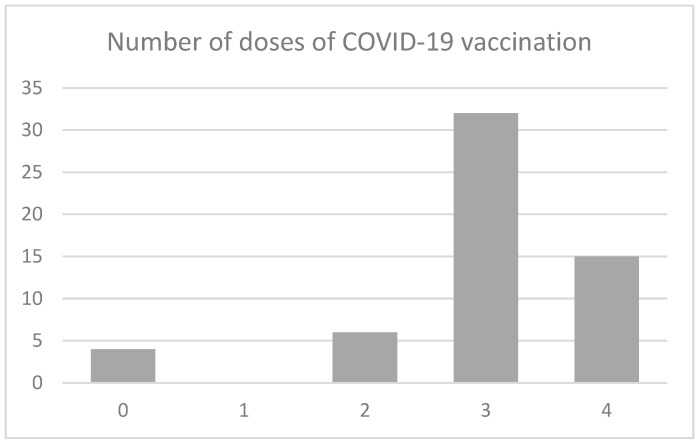
Depiction of received doses of COVID-19 vaccination in patients with SIT. X-axis shows number of doses; Y-axis shows number of patients who received different numbers of doses.

In total, the vaccinated patients received 151 doses of COVID-19 vaccinations. On average, they received three doses of vaccination in total (see Figure A1), and 48 patients (87%) were vaccinated with BioNTech/Pfizer at least once (see Figure 1). Nine patients (16%) received Moderna; eight patients (15%) received Astra Zeneca; and one patient (2%) received the Janssen vaccine (see Figure 1).

### 3.2. More than Half of the Patients (57%) Reported a Reaction After the Administration of COVID-19 Vaccination

**Figure 2:**
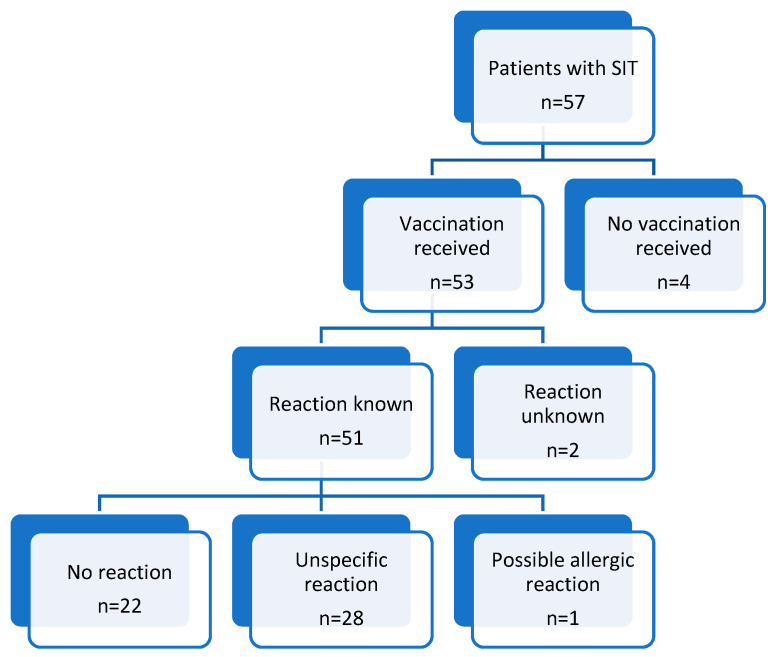
Workup of how many patients received vaccination and how they reacted.

Out of 53 patients, a reaction after vaccination was documented in 51 patients. Of these, 29 (57%) reported an adverse reaction to the vaccine, while the remaining patients did not report any reaction. Of those who experienced a reaction, 1 patient (3%) exhibited possible allergic symptoms, while the remaining 28 patients (97%) reported non-specific illness symptoms (see Figure 2). Adverse reactions occurred in approximately half of the patients at each vaccination, while the remaining patients experienced reactions only at the first or at subsequent timepoints (see Table A2).

### 3.3. Only 1 out of 51 Patients Had a Possible Allergic Reaction After the Administration of COVID-19 Vaccination

A 39-year-old female patient reported the swelling of the hands and lips, as well as paresthesia around the lips, within ten minutes of the first vaccine administration. This corresponds to anaphylactic reaction grade 1, according to Ring and Messmer’s [11] grading scale. Prior to the second dose, prophylactic antihistamine medication was administered. The same vaccine was administered under supervision at a different clinic, where another episode of paresthesia occurred.

The patient had known sensitizations to aeroallergens and oral medication and had previously experienced anaphylaxis of at least grade 2 according to Ring and Messmer’s [11] grading scale. A skin Prick Test was performed following the reactions at our clinic. A potential type-I sensitization to different PEGs or polysorbate 80 could not be verified. The patient was advised to receive the third vaccine according to the recommended schedule and under supervision at our clinic, but the patient was lost to follow-up.

Compared to the other patients, this patient received fewer documented doses of vaccination (see Table 2).

### 3.4. Patients with Adverse Reactions After COVID-19 Vaccination Showed No Significant Differences in Clinical Characteristics Compared to Patients with No Reaction

When we classified our patients into groups with no reaction, an unspecific reaction, or a possible allergic reaction, we could not verify any significant differences in their baseline and serologic characteristics (including tryptase and total IgE levels) or in their atopic comorbidities (see Table 2). Similarly, no higher incidence of adverse reactions was observed in patients with mastocytosis (see Table 2).

HADS questionnaires were used to screen for psychological status in our patients. Although one of our patients with a possible allergic reaction was diagnosed with increased anxiety, no significant differences could be observed between our three patient groups (see Table 2). Patients with no or unspecific reactions had a mean score of 4.6, while those with possible allergic reactions scored 11.0 (see Table 2). Similarly, no significant difference in depression was observed, although one of our patients with a possible allergic reaction met the criteria for a depression diagnosis with a score of 11.0, compared to patients with no reaction (mean score 3.1) and to patients with non-specific reactions (mean score 2.6) (see Table 2). When analyzing our patients’ concerns before receiving each dose of the vaccine, we used a scale from 0 to 4, where 0 represents no concern, and 4 represents severe concern. A significantly higher level of concern was observed in the patient with a possible allergic reaction (median 4.0; *p* < 0.001) compared to the other patients with no reaction (median 0.0, mean 0.7) or an unspecific reaction (median 0.0, mean 0.6) with similar levels of concern. 

Also, no significant differences in the occurrence of no reactions, unspecific reactions, or possible allergic reactions were observed when patients with hymenoptera venom allergy or aeroallergen allergy were compared (*p* = 0.149) (see Table 3). Similarly, no significant difference in the levels of concern could be seen between our two allergy groups (*p* = 0.23).

### 3.5. Previous Adverse Events After Vaccinations and a Sensitization to Aeroallergens Increased the Risk for Reactions to COVID-19 Vaccines

When analyzing prior reactions to various other vaccinations, such as the influenza vaccine, it was observed that our patient, who experienced a possible allergic reaction after the first dose of the COVID-19 vaccination, had also reported allergic reactions after other vaccinations. In contrast, patients who experienced no or only unspecific reactions had no documented history of vaccine-related allergic reactions (see Table 2). Of the patients in the no reaction group, 1 out of 22 had previously experienced a mild unspecific reaction to other vaccinations, as had 9 out of 28 patients (32.1%) in the unspecific reaction group after the COVID-19 vaccination (see Table 2). There were no statistically significant differences in prior anaphylactic reactions between the analyzed reaction groups (see Table 2).

When analyzing the patients with aeroallergies, it was found that they were younger and had a significantly higher number of comorbidities with an atopic background (e.g., asthma and atopic dermatitis), as well as higher total serum IgE levels, compared to patients with hymenoptera allergy (see Table A3). These patients exhibited a significantly higher incidence of reactions to other vaccinations compared to patients with venom allergies. Of the aeroallergen patient group, 8 out of 24 (33%) reported adverse events following other vaccinations, compared to only 11.5% of the hymenoptera venom patient group (*p* = 0.033).

### 3.6. Antihistamine Intake Was Associated with a Significantly Lower Rate of Unspecific Reactions After COVID-19 Vaccination

Of the 21 patients with no reaction after vaccination, 5 (23.8%) had taken antihistamines as a premedication, whereas only 1 (3.6%) of the patients with mild reactions had received premedication (*p* = 0.006; see Table 2). The patient who possibly experienced an allergic reaction after vaccination had received prophylactic treatment with antihistamines.

## 4. Discussion

To our knowledge, this is the first in-depth analysis of the response to the COVID-19 vaccine in patients with severe symptomatic allergies undergoing SIT for aeroallergies and hymenoptera.

The majority of the patients (>90%) that were undergoing SIT at our center had been vaccinated. This rather high rate was surprising given that vaccine hesitancy appears to be common among patients with severe allergies due to early overestimations regarding allergic reactions [14]. We had offered patients allergological advice and the skin Prick Test with PEG in cases where there were doubts of sensitization, which may have helped to address vaccination hesitancy [15]. Comparably, Leru et al. [16] also observed the positive impact of allergist advice on the acceptance of the vaccine. When analyzing the characteristics of the patients who did not receive the vaccine, no significant differences that could have led to vaccine hesitancy were identified. However, Hudson et al. [17] reported younger age as a risk factor for vaccine hesitancy, among other factors.

Later publications reported that the vaccine was well tolerated, even by patients from allergy centers [16] and individuals who had an adverse reaction to the first dose of the vaccine [1].

mRNA vaccines were the first available vaccines in Austria, as reflected by their high application frequency among the different vaccines used. Overall, combinations of different vaccines were less frequent. We did not assess whether the change in these patients was due to availability issues, prior reactions, or patient preferences.

More than half of our patients (57%) reported a reaction after receiving at least one dose of COVID-19 vaccination. A study of medical students found that the rate of adverse reactions following a COVID-19 vaccine was higher than 95% [18]. As in other studies [19], the adverse reactions were mostly mild and unspecific. Injection site pain, fever, body/muscle pain, headache, feeling unwell, and fatigue were the most common adverse events.

Using the Brighton Collaboration Case Definition (BCCD) for anaphylaxis [20], the rate of anaphylaxis to COVID-19 vaccines was very low (approximately 8%), even among patients with suspected allergic reactions who were referred to a Canadian allergy center [19]. Similar results were observed in our cohort, with only one patient experiencing a potential allergic reaction. According to the BCCD, our patient would not have met the criteria for any level of diagnostic certainty. Similarly, Risma et al. [21] reported that IgE-mediated allergies may not be very common due to a lack of reproducibility. However, Filon et al. [22] observed ten times the number of anaphylactic reactions (30%) in patients with high-risk allergies, which included patients with previous anaphylactic reactions to drugs or other vaccines, and drug allergies. Our patient with a possible allergic reaction was a 39-year-old female. Comparably, Blumenthal et al. [23] reported a mean patient age of 41 years and a predominantly female patient group experiencing anaphylaxis following vaccination.

When comparing the patients who had not reported any reactions with those who had reported unspecific reactions or possible allergic reactions, we found no significant differences in terms of age, sex, type of allergy/SIT, or serum values such as total IgE or tryptase levels (see Table 2). However, a history of anaphylaxis was observed in the patient with possible allergic reactions, and a high rate of unspecific reactions to other vaccines was observed in the patients with reactions to the COVID-19 vaccines, as previously reported by Filon et al. [22]. There were no differences in atopic comorbidities or anamnesis of anaphylaxis to hymenoptera venom or other allergens between the subgroups of patients who had a reaction to the vaccine. Neither inhalant allergies nor allergic comorbidities could be identified as risk factors, nor could venom allergies [22]. Zurek et al. [24] assessed the occurrence of anaphylactic reactions in patients with hymenoptera allergies and found no elevated numbers compared to the general population.

Interestingly, our patient who experienced a possible allergic reaction reported significantly greater concerns prior to receiving the COVID-19 vaccine compared to patients who experienced no or unspecific reactions. Since the patients were asked about their concerns months after vaccination, it could also be hypothesized that these concerns arose as a result of the reactions. However, anxiety and concerns can be an important factor in vaccine hesitancy [15]. Additionally, it was observed that anxiety-related reactions were mistakenly identified as allergic reactions by patients or other individuals without a medical background [16].

The rate at which patients took antihistamines prior to receiving the COVID-19 vaccination was low (13%), but this was significantly associated with the absence of adverse events after vaccination. Previously, antihistamine intake was reported by Teufelberger et al. [25] as being protective.

This study has several limitations, as it was designed as an exploratory project with a small sample size and a single-center study design. Furthermore, most patients completed the questionnaires retrospectively, with recall periods of several weeks or even months. The questionnaires used self-report tools and did not require clinical supervision—accordingly, the reports may lack sufficient clinical relevance. Moreover, our patient group consisted of patients with a mean age of 43 years, whereas other age groups, such as children, were not included. Also, there is no comparison group without allergies/anaphylaxis. However, reports on vaccine tolerance in patient cohorts with a history of anaphylaxis are rare. This study can contribute to future studies on this aspect and provide clinical guidance for patients regarding future upcoming (mRNA) vaccines.

## 5. Conclusions

In our patient cohort of those with severe allergies undergoing SIT, the safety of the COVID-19 vaccine was demonstrated, with no severe adverse or anaphylactic reactions observed. Mild, unspecific reactions were frequent, and taking antihistamines may help to reduce side effects after COVID-19 vaccinations in patients with allergies. In order to further evaluate this hypothesis, it is necessary to conduct prospective studies on this topic.

## Figures and Tables

**Table 1:** Characteristics of patients undergoing SIT. Results are shown as median (IQR) and mean ± SD. Abbreviations: y—years; SD—standard deviation; IQR—interquartile range; m—male; f—female.

Characteristics	Patients with SIT (*n* = 57)
Age	
Median (IQR)	43.0 (28.0)
Mean ± SD	46.2 ± 16.8
Sex (m:f)	33:24
Mastocytosis, *n* (%)	3 (5.3%)
Number of pos. Allergy Categories *	
Median (IQR)	1.0 (1.0)
Mean ± SD	1.4 ± 0.7
Prior Anaphylaxis, *n* (%)	34 (59.6%)

* Allergy Categories: aeroallergies, food allergies, contact allergies, venom allergies, allergies to injectable drugs, and allergies to oral medication.

**Table 2:** Cohort characteristics.

	No Reaction	Unspecific Reaction	Possible Allergic Reaction	Significance
	(*n* = 22)	(*n* = 28)	(*n* = 1)	*p*-Value
Basic characteristics				
Number of vaccinations				*0.099*
Median (IQR)	*3.0 (1.0)*	*3.0 (0.0)*	*2.0*	
Mean ± SD	*3.3 ± 0.6*	*3.1 ± 0.6*		
Age (y)				
Median (IQR)	54.0 (22.0)	40.5 (28.8)	39.0	0.199
Mean ± SD	51.6 ± 15.6	43.8 ± 17.2		
Sex (m:f)	13:9	17:11	0:1	0.479
Allergy group				0.278
Hymenoptera venom	14 (63.3%)	13 (46.4%)	0 (0%)	
Aeroallergen	8 (36.65%)	15 (53.6%)	1 (100%)	
Serology				
Total IgE				0.43
Median (IQR)	94.0 (352.3)	135.0 (175.0)	332.0	
Mean ± SD	199.7 ± 187.6	266.6 ± 669.5		
Tryptase	(*n* = 21)	(*n* = 27)		
Median (IQR)	5.1 (3.2)	5.2 (3.9)	4.1	0.857
Mean ± SD	6.2 ± 6.1	7.1 ± 5.8		
Comorbidities				
Allergic rhinitis	9 (40.9%)	18 (64.3%)	1 (100%)	0.169
Asthma	4 (18.2%)	5 (17.9%)	0 (0%)	0.896
Atopic dermatitis	1 (4.5%)	5 (17.9%)	0 (0%)	0.326
Urticaria	2 (9.1%)	1 (3.6%)	0 (0%)	0.69
Mastocytosis	1 (4.5%)	2 (7.1%)	0 (0%)	0.899
Anxiety	(*n* = 19)			0.331
Median (IQR)	5.0 (5.0)	4.0 (3.8)	11.0	
Mean ± SD	4.6 ± 3.3	4.6 ± 3.2		
Depression	(*n* = 19)			0.229
Median (IQR)	2.0 (5.0)	2.0 (3.0)	11.0	
Mean ± SD	3.1 ± 2.9	2.6 ± 2.9		
Concerns before vaccination				**<0.001**
Median (IQR)	**0.0 (1.3)**	**0.0 (1.0)**	**4.0**	
Mean ± SD	**±0.8**	**0.6 ± 0.8**		
COVID-19 infection before vaccination	(*n* = 21)	(*n* = 27)		0.725
	2 (9.5%)	4 (14.8%)	0 (0%)	
Prior reactions to medication				
Anamnestic allergic reactions after oral medication	*4 (18.2%)*	*3 (10.7%)*	*1 (100%)*	*0.05*
Anamnestic allergic reactions after injectable medication	0 (0%)	1 (3.6%)	0 (0%)	0.658
Anamnestic unspecific reactions to other vaccines (e.g., influenza)		(*n* = 27)		
	**1 (4.5%)**	**9 (33.3%)**	**0 (0%)**	**<0.001**
Prior anaphylaxis				
Any prior anaphylaxis	15 (68.2%)	13 (46.4%)	1 (100%)	0.213
Anamnestic anaphylaxis to other vaccines (e.g., influenza)	**0**	**0**	**1 (100%)**	**<0.001**
Premedication				
Prophylactic medication with antihistamines	(*n* = 21) **5 (23.8%)**	**1 (3.6%)**	**1 (100%)**	**0.006**

No reaction group: patients with no reaction to COVID-19 vaccination. Unspecific reaction group: patients with unspecific reactions (e.g., fever, limb pain, local reaction) to COVID-19 vaccination. Possible allergic reaction group: patients with possible allergic reactions (e.g., urticaria, pruritus, dyspnea) to COVID-19 vaccination. Results are shown as median ± IQR of indicated number of individual data points or independent experiments. Results are grouped into six categories, named and shown on blue background. Abbreviations: y—years; SD—standard deviation; IQR—interquartile range; m—male; f—female. Kruskal–Wallis test was used for age, number of allergies, total IgE and tryptase levels, and hospital anxiety and depression scale score and Chi-square test for other assessments. *p*-values < 0.05 were considered as statistically significant. Significant values are displayed in bold, trends (*p* < 0.1) in italic.

**Table 3:** Reactions in patients of hymenoptera allergy group and patients of aeroallergy group. Kruskal–Wallis test was performed for comparison. *p*-values < 0.05 were considered as statistically significant.

	Patients in Hymenoptera Allergy Group (*n* = 27)	Patients in Aeroallergy Group (*n* = 24)	Significance *p*
No reaction	14 (51.9%)	8 (33.3%)	0.201
Unspecific reaction	13 (48.1%)	15 (62.5%)	0.705
Possible allergic reaction	0 (0%)	1 (4.2%)	-

## Data Availability

The data that support the findings of this study are available from the corresponding author upon reasonable request.

## Abbreviations

The following abbreviations are used in this manuscript:

COVID-19	Corona Virus Disease 2019
f	Female
IgE	Immunoglobulin E
IQR	Interquartile Range
HADS	Hospital Anxiety and Depression Scale
m	Male
PEG	Polyethylene Glycol
SIT	Specific Immunotherapy
SD	Standard Deviation
y	Years

## Appendix A

**Table A1:** Cohort characteristics of vaccinated and non-vaccinated patients. Vaccinated group: patients with SIT who received COVID-19 vaccination. Not vaccinated group: patients with SIT who did not receive COVID-19 vaccination. Results are shown as median ± IQR of indicated number of individual data points or independent experiments. Abbreviations: y—years; SD—standard deviation; IQR—interquartile range; m—male; f—female. Kruskal–Wallis test was used for age, number of allergies, total IgE and tryptase levels, and hospital anxiety and depression scale score and Chi-square test for other assessments. *p*-values < 0.05 were considered as statistically significant. Significant values are displayed in bold.

	Vaccinated (*n* = 53)	Not Vaccinated (*n* = 4)	Significance *p*-Value
Basic characteristics			
Age (y)			
Median (IQR)	43.0 (27.0)	41.0 (37.8)	0.69
Mean ± SD	46.4 ± 16.7	43.8 ± 20.5	
Sex (m:f)	31:22	2:2	1.0
Mastocytosis	3 (5.7%)	0 (0%)	1.0
Number of different allergy categories			0.25
Median (IQR)	1.0 (1.0)	1.0 (0.0)	
Mean ± SD	1.5 ± 0.8	1.0 ± 0.0	
Aeroallergies	**32 (60.4%)**	**0 (0%)**	**0.03**
Food allergies	6 (11.3%)	0 (0%)	1.0
Contact allergies	1 (1.9%)	0 (0%)	1.0
Allergies to oral medication	8 (15.1%)	0 (0%)	1.0
Allergies to injectables	1 (1.9%)	0 (0%)	1.0
Venom allergies	30 (56.6%)	4 (100%)	0.14
Prior anaphylaxis	30 (56.6%)	4 (100%)	0.14
Serology			
Total IgE			0.18
Median (IQR)	138.0 (199.0)	60.0 (0.0)	
Mean ± SD	243.8 ± 526.2	58.3 ± 36.5	
Tryptase			0.61
Median (IQR)	5.2 (3.1)	6.4 (0.0)	
Mean ± SD	6.8 ± 5.9	5.9 ± 1.8	
Comorbidities			
Allergic rhinitis	**30 (56.6%)**	**0 (0%)**	**0.04**
Asthma	9 (17.0%)	0 (0%)	1.0
Atopic dermatitis	6 (11.3%)	0 (0%)	1.0
Urticaria	3 (5.7%)	0 (0%)	1.0
Anxiety			0.59
Median (IQR)	4.0 (5.0)	4.0 (0.0)	
Mean ± SD	4.8 ± 3.3	5.7 ± 2.9	
Depression			0.51
Median (IQR)	2.0 (4.0)	5.0 (0.0)	
Mean ± SD	3.0 ± 3.1	5.3 ± 2.5	

**Table A2:** Occurrence of adverse reactions depending on number of doses. Results are shown as number and percentage.

	No Reaction	Adverse Reaction *n* = 29		
		Reaction After 1 Dose, Then No Reaction	No Reaction After 1 Dose, Then Reaction	Reaction After 1 and Other Doses
Number of Patients	22	7 (24.1%)	7 (24.1%)	15 (51.7%)

**Table A3:** Cohort characteristics of subgroups of patients according to their current ongoing SIT. Hymenoptera allergy group: patients who received SIT because of hymenoptera allergy. Aeroallergy group: patients who received SIT because of aeroallergy. Results are shown as median ± IQR of indicated number of individual data points or independent experiments. Abbreviations: y—years; SD—standard deviation; IQR—interquartile range; m—male; f—female. Kruskal–Wallis test was used for age, number of allergies, total IgE and tryptase levels, and anxiety and depression score and Chi-square test for other assessments. *p*-values < 0.05 were considered as statistically significant. Significant values are displayed in bold, trends (*p* < 0.1) in italic.

	Both Groups	Hymenoptera Allergy	Aeroallergies	Significance
	(*n* = 51)	(*n* = 27)	(*n* = 24)	*p*-Value
Basic characteristics				
Number of vaccinations				0.333
Median (IQR)	3.0 (1.0)	3.0 (1.0)	3.0 (0.8)	
Mean ± SD	3.2 ± 0.6	3.3 ± 0.6	3.1 ± 0.7	
Reaction after vaccination				0.149
(unspecific/possibly allergic/no reaction)	28:1:22	13:0:14	15:1:8	
Age (y)				**<0.001**
Median (IQR)	46.0 (27.0)	**54.0 (23.0)**	**34.5 (17.8)**	
Mean ± SD	46.2 ± 16.8	**54.4 ± 14.1**	**38.7 ± 15.6**	
Sex (m:f)	30:21	16:11	14:10	0.984
Mastocytosis	3 (5.9%)	3 (11.1%)	0 (0%)	0.248
Number of different allergy categories				0.687
Median (IQR)	1.0 (1.0)	1.0 (1.0)	1.0 (1.0)	
Mean ± SD	1.5 ± 0.8	1.5 ± 0.8	1.4 ± 0.7	
Aeroallergies	30 (58.8%)	**6 (22.2%)**	**24 (100%)**	**<0.001**
Food allergies	5 (9.8%)	2 (7.4%)	3 (12.5%)	0.643
Contact allergies	1 (2.0%)	1 (3.7%)	0 (0%)	1.000
Allergies to oral medication	8 (15.7%)	4 (14.8%)	4 (16.7%)	0.718
Allergies to injectables	1 (2.0%)	1 (3.7%)	0 (0%)	1.000
Venom allergies	30 (58.8%)	**27 (100%)**	**3 (12.5%)**	**<0.001**
Prior anaphylaxis	29 (56.9%)	**25 (92.6%)**	**4 (16.7%)**	**<0.001**
Comorbidities				
Allergic rhinitis	28 (54.9%)	**4 (14.8%)**	**24 (100%)**	**<0.001**
Asthma	9 (17.6%)	**1 (3.7%)**	**8 (33.3%)**	**0.007**
Atopic dermatitis	6 (11.8%)	**0 (0%)**	**6 (25.0%)**	**0.005**
Urticaria	3 (5.9%)	2 (7.4%)	1 (4.2%)	1.000
Anxiety				0.575
Median (IQR)	4.0 (4.75)	3.0 (5.0)	4.0 (3.0)	
Mean ± SD	4.7 ± 3.3	4.4 ± 3.4	5.0 ± 3.2	
	(*n* = 48)	(*n* = 25)	(*n* = 23)	
Depression				0.373
Median (IQR)	2.0 (4.0)	1.0 (4.5)	2.0 (4.0)	
Mean ± SD	3.0 ± 3.1	2.6 ± 2.8	3.4 ± 3.4	
	(*n* = 48)	(*n* = 25)	(*n* = 23)	
COVID-19 infection (before/after/both/no)	2:21:4:22	1:11:1:12	1:10:3:10	0.350
	(*n* = 49)	(*n* = 25)		
Serology				
Total IgE				**0.006**
Median (IQR)	132.0 (199.0)	**70.0 (118.0)**	**203.0 (324.5)**	
Mean ± SD	239.1 ± 508.1	**123.1 ± 137.3**	**369.5 ± 711.6**	
Tryptase				*0.075*
Median (IQR)	5.1 (3.1)	*5.7 (5.4)*	*4.1 (2.7)*	
Mean ± SD	6.7 ± 5.9	*8.2* ± *7.3*	*4.7* ± *2.0*	
	(*n* = 49)		(*n* = 22)	
Prior Vaccinations				
Reactions to other vaccines (e.g., influenza)	10:1:39	**3:0:23**	**7:1:16**	**0.033**
(unspecific/possibly allergic/no reaction)	(*n* = 50)	(*n* = 26)		
Concerns before vaccination				0.300
Median (IQR)	0.0 (1.0)	0.0 (1.0)	1.0 (1.8)	
Mean ± SD	0.7 ± 0.9	0.9 ± 1.0	0.9 ± 1.0	
Prophylactic medication with antihistamines	7 (13.7%)	5 (18.5%)	2 (8.7%)	0.430
			(*n* = 23)	
